# Morphologic and Genetic Characterization of *Protospirura canariensis* n. sp. (Nematoda, Spiruridae), a Parasite of the Black Rat *Rattus rattus* (Rodentia, Muridae) from El Hierro Island (Canary Archipelago, Spain) ^†^

**DOI:** 10.3390/ani13111806

**Published:** 2023-05-30

**Authors:** Jordi Miquel, Natalia Martín-Carrillo, Alexis Ribas, Santiago Sánchez-Vicente, Carlos Feliu, Pilar Foronda

**Affiliations:** 1 Secció de Parasitologia, Departament de Biologia, Sanitat i Medi Ambient, Facultat de Farmàcia i Ciències de l’Alimentació, Universitat de Barcelona, Avgda. Joan XXIII, sn, 08028 Barcelona, Spain; aribas@ub.edu (A.R.); cfeliu@ub.edu (C.F.); 2 Institut de Recerca de la Biodiversitat (IRBio), Universitat de Barcelona, Avgda. Diagonal, 645, 08028 Barcelona, Spain; 3 Instituto Universitario de Enfermedades Tropicales y Salud Pública de Canarias, Universidad de La Laguna, Avda. Astrofísico F. Sánchez, sn, 38203 La Laguna, Canary Islands, Spain; nmartinc@ull.edu.es (N.M.-C.); pforonda@ull.edu.es (P.F.); 4 Center for Infection and Immunity, Mailman School of Public Health, Columbia University, 722 West 168th Street, New York, NY 10032, USA; sanchez.v.santi@gmail.com; 5 Departamento Obstetricia y Ginecología, Pediatría, Medicina Preventiva y Salud Pública, Toxicología, Medicina Legal y Forense y Parasitología, Facultad de Farmacia, Universidad de La Laguna, Avda. Astrofísico F. Sánchez, sn, 38203 La Laguna, Canary Islands, Spain

**Keywords:** *Protospirura canariensis* n. sp., Spiruridae, Nematoda, *Rattus rattus*, Muridae, Canary Islands

## Abstract

**Simple Summary:**

A new spirurid nematode, *Protospirura canariensis* n. sp., a parasite of the black rat in El Hierro (Canary Islands, Spain), was described by means of light and scanning electron microscopy. The morphological characteristics of the new species and the existing *Protospirura* species, as well as their host species and geographical distributions, were useful criteria in distinguishing *P. canariensis* n. sp. from the remaining species of the genus. Novel molecular phylogenetic data of the new nematode were obtained, and were compared with those from available related *Protospirura* species.

**Abstract:**

A new spirurid nematode, *Protospirura canariensis* n. sp., a parasite of the black rat *Rattus rattus* Linnaeus, 1758 (Rodentia: Muridae), in El Hierro Island (Canary Archipelago, Spain), was described by means of light (LM) and scanning electron microscopy (SEM). The most discriminating characteristics between the new species and the remaining species of the genus *Protospirura* were observed to be the following: (a) the number of tooth-like outgrowths in the sub-median and lateral lobes of the pseudolabia, both in males and females (2 and 4, respectively); (b) the size of the right and left spicules in males (643–715 µm and 309–412 µm, respectively); and (c) the numbers and arrangements of the cloacal papillae in males. The new species has a total of 17 cloacal papillae (4 large and pedunculated pairs of precloacal papillae, an unpaired precloacal papilla and 4 pairs of postcloacal papillae). The arrangement of the postcloacal papillae are as follows: the first pair are large, pedunculated and placed near the posterior edge of cloaca; the three remaining postcloacal pairs are grouped and located near the posterior tip. In the latter group, the papillae in the first pair are large and pedunculated. The parasitized hosts and their geographical distributions were also useful criteria in distinguishing *P. canariensis* n. sp. from the remaining species of the genus *Protospirura*. In addition, the cytochrome c oxidase subunit I (cox1) sequence of the new species was obtained and compared with the available data of related species.

## 1. Introduction

The taxonomic status of the cosmopolitan genus *Protospirura* Seurat, 1914 (Spiruridae), has been confusing for a long time, particularly due to the morphological similitudes with the genus *Mastophorus* Diesing, 1853 (Spirocercidae). According to several authors [1,2], this confusion is due to the consideration of inappropriate characters. In this sense, Chitwood [1] established the following differential characteristics between the genera *Protospirura* and *Mastophorus*: the number of teeth in the pseudolabia (two or four in *Protospirura* vs. three, five, seven or nine in *Mastophorus*); the morphology of the pharynx (laterally compressed in *Protospirura* vs. cylindrical in *Mastophorus*); the morphology of cloacal papillae in males (sessile in *Protospirura* vs. pedunculated in *Mastophorus*); the tail length in males (short in *Protospirura* vs. long in *Mastophorus*); and the position of the vulva in females (post-equatorial in *Protospirura* vs. pre-equatorial in *Mastophorus*). However, some of these characteristics are not present in all of the currently accepted species of the genus *Protospirura*. In fact, some *Protospirura* species have pedunculated cloacal papillae [3,4,5,6,7], or the vulva is located anteriorly to the mid-body [7,8,9]. Furthermore, the morphology of the pseudolabia in genera *Protospirura* vs. *Mastophorus* is quite different. In the *Protospirura* genus, the four sub-median lobes are clearly less developed than the lateral lobes, whereas in the *Mastophorus* genus, the oral opening is hexagonal and the sub-median lobes of the pseudolabia are well-developed and quadrangular [10].

Currently, the genus *Protospirura* comprises 13 parasitic species of mammals included in 5 orders and in 18 families: Artiodactyla (Bovidae), Carnivora (Canidae, Felidae and Viverridae), Eulipotyphla (Erinaceidae and Talpidae), Primates (Aotidae, Atelidae, Cebidae, Cercopythecidae, Hominidae and Lorisidae) and Rodentia (Bathyergidae, Cricetidae, Heteromyidae, Muridae, Nesomyidae and Sciuridae) [3,4,5,6,7,8,9,11,12,13,14,15,16,17,18,19]. Except for *P. muricola* Gedoelst, 1916, which has a worldwide distribution including Africa, the Caribbean region, Central and South America, Southeast Asia and Europe [15], the remaining species of this genus have a limited geographical distribution. With respect to the parasitized hosts, only seven species of *Protospirura* have been recorded in Muridae rodents. These are *P. armeniana* Ajojan, 1951; *P. chabaudi* Vuylsteke, 1964; *P. kaindiensis* Smales, 2001; *P. munimuniensis* Smales, 2021; *P. muricola*, *P. okinavensis* Hasegawa, 1990; and *P. siamensis* Ribas, Veciana, Chaisiri and Morand, 2012 [8,9,14,15,16,17,18,19]. Other species have been described as belonging to the genus *Protospirura*, namely *P. ascaroidea* Hall, 1916; *P. bestiarum* Kreis, 1953; *P. columbiana* Cram, 1926; *P. glareoli* Soltys, 1949; *P. gracilis* Cram, 1924; *P. labiodenta* Linstow, 1899; and *P. marsupialis* Baylis, 1934. These were considered synonyms of *Mastophorus muris* (Gmelin, 1790) by Chitwood [1]. These synonymies were further confirmed by Wertheim [2]. Additionally, other species described posteriorly, namely *P. chanchanensis* Ibáñez, 1966; *P. paucidentata* Wang, Zhao and Ching, 1978; and *P. srivastavai* Gupta and Trivedi, 1987, should be included in the Spirocercidae because their pharynx is not laterally compressed [8]. Finally, *P. bonnei* Ortlepp, 1924 was considered a synonym of *P. muricola* and *P. congolense* Vuylsteke, 1956 was transferred to the genus *Mastophorus* by Quentin [20].

In the present study, we describe a new species, *Protospirura canariensis* n. sp., which parasitizes the black rat, *Rattus rattus* Linnaeus, 1758 (Rodentia, Muridae), in El Hierro Island (Canary Archipelago, Spain). Additionally, the sequence of the mitochondrial cytochrome c oxidase subunit I gene (cox1) was provided and compared with the data of related species.

## 2. Materials and Methods

### 2.1. Specimens

**Figure 1:**
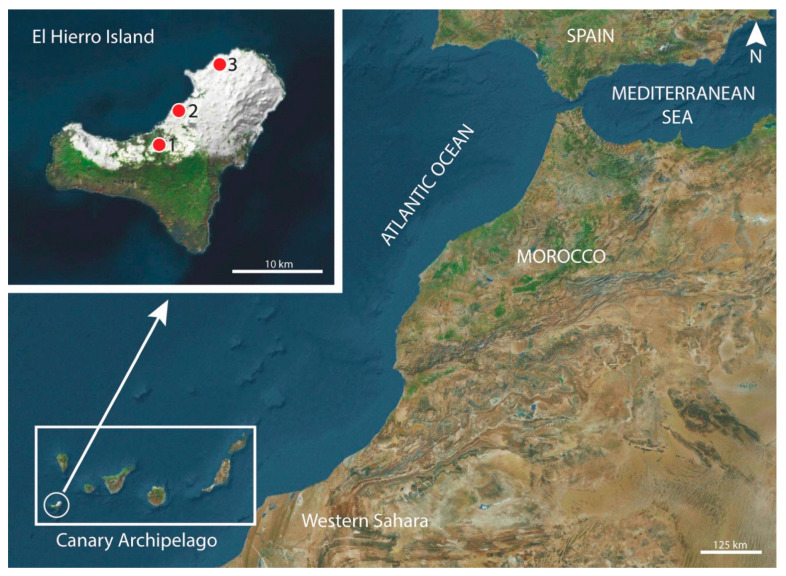
Localities where *Rattus rattus* are parasitized by *Protospirura canariensis* n. sp. were captured on El Hierro Island (Canary Archipelago, Spain). (1) Camino-Frontera (27°44′38.07″ N, 18°2′25.4″ W); (2) Lagartario-Frontera (type locality) (27°46′29.9″ N, 17°59′55.59″ W); (3) Túnel-Valverde (27°49′12.41″ N, 17°57′49.27″ W).

Nematodes were recovered from the stomach of several black rats *Rattus rattus* captured in Lagartario-Frontera (27°46′29.9″ N, 17°59′55.59″ W), Camino-Frontera (27°44′38.07″ N, 18°2′25.4″ W) and Túnel-Valverde (27°49′12.41″ N, 17°57′49.27″ W) (El Hierro Island, Canary Archipelago, Spain) (Figure 1) during several trapping campaigns in 2008, 2009 and 2010. The studied rats were captured using Sherman traps (H.B. Sherman Traps, Inc., Tallahassee, FL, USA) and wire-mesh traps of the Manufrance type (Saint-Étienne, France) or Firobind type (Besançon, France). The animals were sacrificed using cervical dislocation, and then examined for gastrointestinal helminths under a stereomicroscope.

### 2.2. Light Microscopy Study

The specimens were mounted in Amann lactophenol on slides, and then observed under the light microscope (LM) Leica DMLB (Leica Microsystems, Wetzlar, Germany). Drawings were made with the aid of a drawing tube, and later modified using Adobe Illustrator software (Adobe, San José, CA, USA). All of the measurements are specified in micrometers, except where indicated.

### 2.3. Scanning Electron Microscopy Study

Some nematodes (three males and six females) were preserved for scanning electron microscopy (SEM) examination. Initially, they were fixed in 70% ethanol in the field; later in the laboratory, they were dehydrated in an ethanol series and critical-point dried with carbon dioxide (Emitech K850X, Quorum Technologies Ltd., Laughton, East Sussex, UK). Finally, the worms were mounted on stubs with conductive adhesive tape and colloidal silver, coated with carbon in an Emitech K950X evaporator (Quorum Technologies Ltd.), and examined using a JSM-7001F field emission scanning electron microscope (JEOL Ltd., Tokyo, Japan) operated at an accelerating voltage of 10 kV in the “Centres Científics i Tecnològics de la Universitat de Barcelona (CCiTUB)”.

### 2.4. Molecular Analyses and Phylogenetic Tree

The genomic DNA was extracted using fragments of *Protospirura canariensis* n. sp. specimens that had been reserved for this purpose. A total of 10 nematode fragments were extracted. The fragments were deposited in tubes containing a mixture of 250 µL of a lysis solution composed of 30 mM Tris-HCL pH 8.0, mM EDTA and 0.4% SDS. In addition, 3 µL of proteinase K (20 mL^−1^) (PanReac AppliChem ITW Reagents, Darmstadt, Germany) was added, and then the tubes incubated at 56 °C overnight. The following day, 250 µL of NH_4_Ac 4 M was added, mixed thoroughly and subsequently incubated for 30 min at room temperature (15 °C–25 °C). The mix was spun for 10 min at 13,000 rpm, and the pellet was discarded. The DNA was then precipitated from the supernatant with ethanol, and the pellet was resuspended in 200 µL of 1X TE (10 mM Tris-HCL pH 8.1 mM EDTA) [21]. The quantity and quality of genomic DNA was checked using a DeNovix DS-11 + spectrophotometer (DeNovix Inc., Wilmington, DE, USA).

PCR screening of the DNA was based on cytochrome c oxidase subunit I (cox1) using the primers COIIntF and COIIntR described by Gaillard et al. [22]. The PCR amplification contained 1X Buffer (VWR), 0.2 mM of each dNTP (VWR), 1.5 mM MgCl_2_ (VWR), 20–40 ng of total genomic DNA in a total volume of 50 µL. The amplification was conducted with an XP Cycler (Hangzhou Bioer Technology Co. Ltd., Hangzhou, China) under the following conditions: 94 °C for 3 min; 35 cycles at 94 °C for 30 s, 54 °C for 30 s, 68 °C for 45 s; and a final extension at 68 °C for 10 min [22]. The resulting amplifications were visualized on 1.5% agarose gel at 90 V for 1 h.

The PCR products that presented the expected size (650 bp) were sequenced at Macrogen Spain Inc. (Madrid, Spain) with primers COIIntF/COIIntR [22]. The sequences that were obtained using the Sanger method were interpreted with MEGA X software [23], using the multiple alignment program ClustalW included in MEGA X, and minor corrections were made by hand. The sequences were subsequently analyzed with the basic local alignment search tool (BLAST), and their identities confirmed via homology comparison.

Phylogenetic relationship analyses based on the maximum likelihood method were carried out with the p distance and Kimura 2-parameter model [24], and with 1000 bootstrap replications based on the cytochrome c oxidase subunit I (cox1) gene sequences, in order to explore the relationships among the species. The sequence *Schistosoma mansoni* Sambon, 1907 NC002545 was used as the outgroup.

## 3. Results

### 3.1. Taxonomic Summary

Family Spiruridae Oerley, 1885

Genus *Protospirura* Seurat, 1914

**Figure 2:**
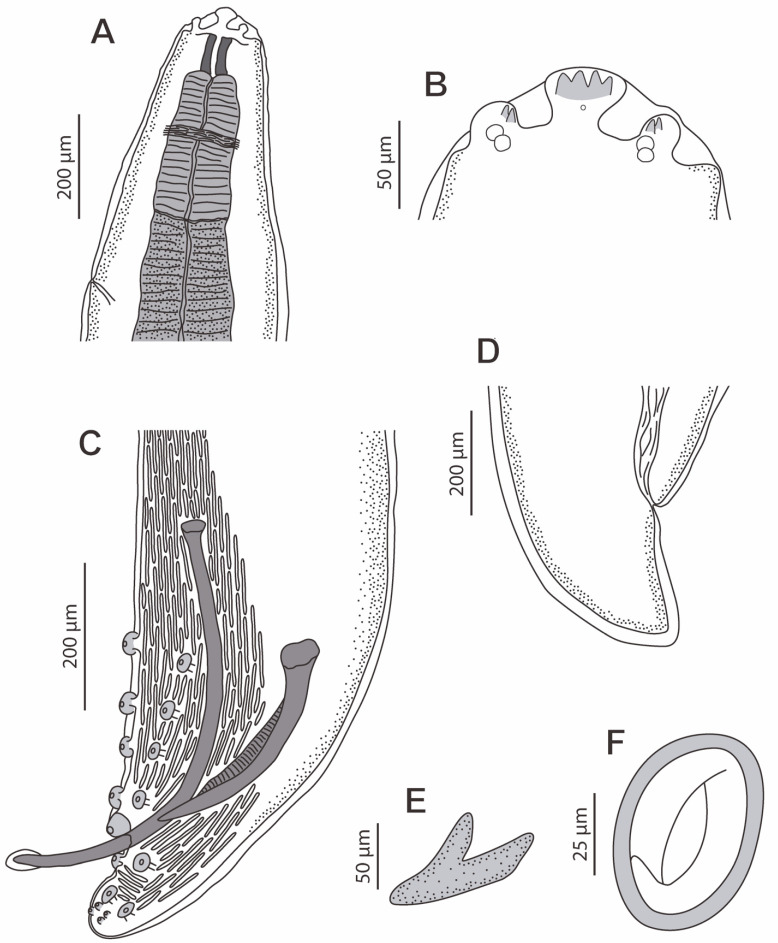
*Protospirura canariensis* n. sp., male and female. (**A**) Cephalic end of female, lateral view. (**B**) Sub-median and lateral lobes of one of the pseudolabia of a female, lateral view. (**C**) Caudal end of a male showing the two spicules and the cloacal papillae, lateral view. (**D**) Tail of a female, lateral view. (**E**) V-shaped gubernaculum. (**F**) Embryonated egg.

**Figure 3:**
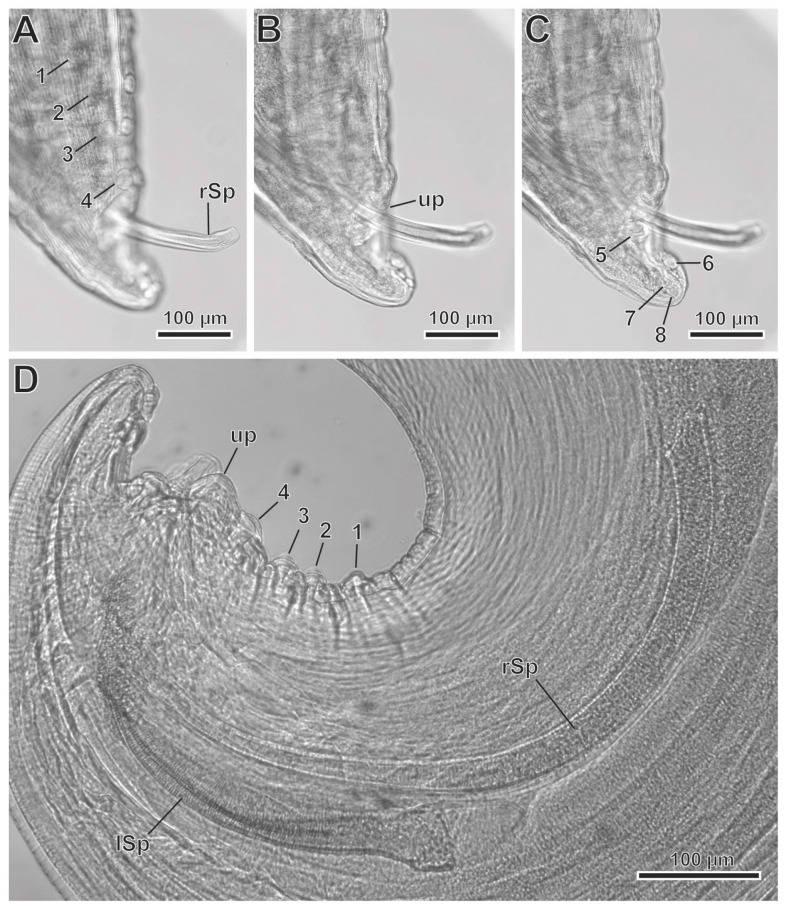
*Protospirura canariensis* n. sp., male, LM. (**A**) Caudal end showing the four pairs of precloacal papillae (1 to 4). (**B**) Caudal end showing the unpaired precloacal papilla (up). (**C**) Caudal end showing the four pairs of postcloacal papillae (5 to 8). (**D**) Right (rSp) and left spicules (lSp).

**Figure 4:**
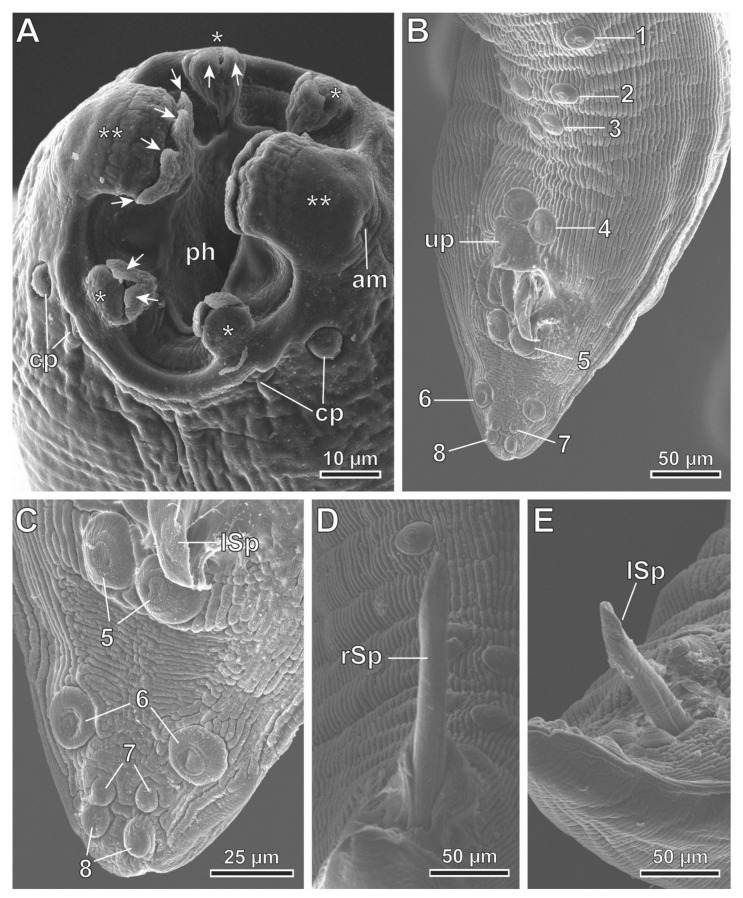
*Protospirura canariensis* n. sp., male, SEM. (**A**) Apical view of the cephalic end showing the two tri-lobed pseudolabia. (**B**) Caudal end illustrating the distribution of cloacal papillae. (**C**) Postcloacal pairs of papillae. (**D**) Right spicule (rSp). (**E**) Left spicule (lSp). (arrows) tooth-like outgrowths; (*) sub-median lobes; (**) lateral lobes; (1–4) pairs of precloacal papillae; (5–8) pairs of postcloacal papillae; (am) amphid; (cp) cephalic papillae; (ph) pharynx; (up) unpaired precloacal papilla.

**Figure 5:**
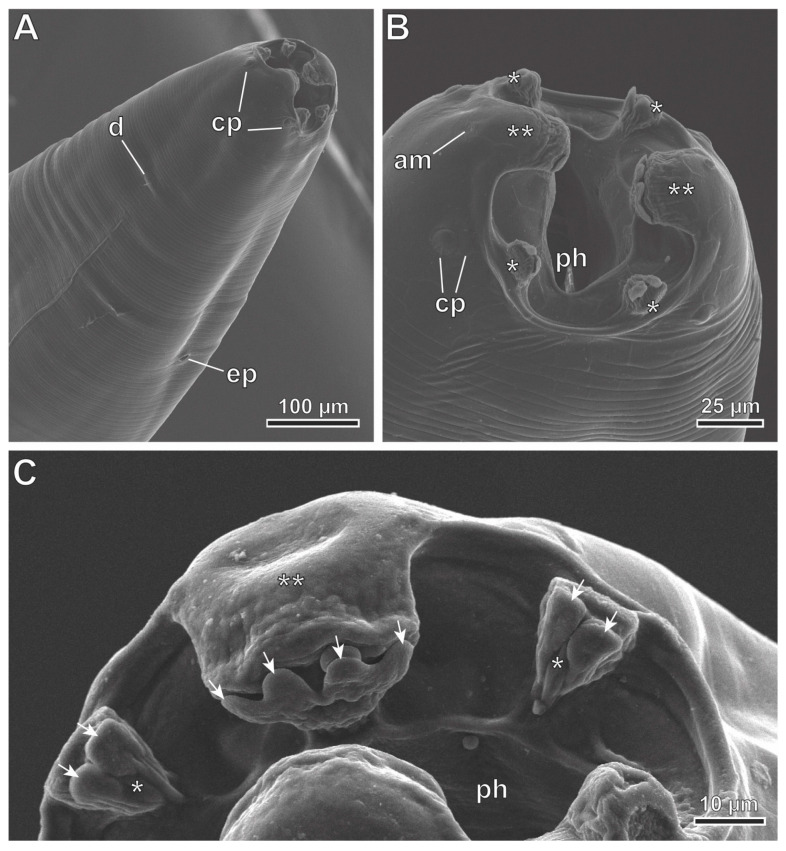
*Protospirura canariensis* n. sp., female, SEM. (**A**) Lateroventral view of the cephalic end showing the right deirid (d) and the excretory pore (ep). (**B**) Apical view of the cephalic end showing the two tri-lobed pseudolabia. (**C**) One of the pseudolabia showing tooth-like outgrowths (arrows). (*) sub-median lobes; (**) lateral lobes; (am) amphid; (cp) cephalic papillae; (ph) pharynx.

**Figure 6:**
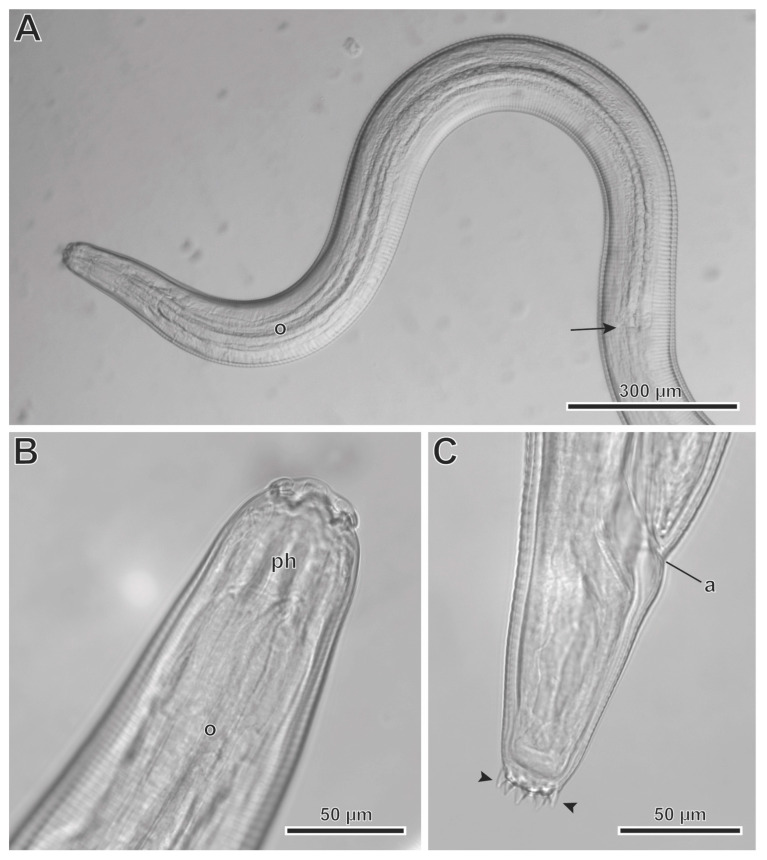
*Protospirura canariensis* n. sp., L3 larva, LM. (**A**) Cephalic end showing the entire esophagus (o). (**B**) Cephalic end showing the pharynx (ph). (**C**) Notched posterior end showing caudal pointed outgrowths (arrowheads). (arrow) posterior extremity of the esophagus; (a) anus.

*Protospirura canariensis* n. sp. (Figure 2A–F, Figure 3A–D, Figure 4A–E, Figure 5A–C and Figure 6A–C)

Type host: *Rattus rattus* Linnaeus, 1758 (Rodentia: Muridae).

Type locality: Lagartario-Frontera (El Hierro Island, Canary Archipelago, Spain) (27°46′29.9″ N, 17°59′55.59″ W) (Figure 1).

Other localities: Camino-Frontera (El Hierro Island, Canary Archipelago, Spain) (27°44′38.07″ N, 18°2′25.4″ W), Túnel-Valverde (El Hierro Island, Canary Archipelago, Spain) (27°49′12.41″ N, 17°57′49.27″ W) (Figure 1).

Site of infection: stomach.

The type specimens were deposited in the “Muséum National d’Histoire Naturelle” (Paris, France) under the following accession numbers:

- holotype, ♂No. 1, MNHN HEL1905.
- allotype, ♀No. 1, MNHN HEL1906.
- Nineteen paratypes:
  - Seven males: ♂No. 2, MNHN HEL1907; ♂No. 4, MNHN HEL1908; ♂No. 5, MNHN HEL1909; ♂No. 7, MNHN HEL1910; ♂No. 8, MNHN HEL1911; ♂No. 10, MNHN HEL1912 and ♂No. 11, MNHN HEL1913.
  - Twelve females: ♀No. 2, MNHN HEL1914; ♀No. 3, MNHN HEL1915; ♀No. 4, MNHN HEL1916; ♀No. 5, MNHN HEL1917; ♀No. 6, MNHN HEL1918; ♀No. 7, MNHN HEL1919; ♀No. 8, MNHN HEL1920; ♀No. 9, MNHN HEL1921; ♀No. 10, MNHN HEL1922; ♀No. 11, MNHN HEL1923; ♀No. 12, MNHN HEL1924 and ♀No. 13, MNHN HEL1925.

The mitochondrial cytochrome c oxidase subunit I gene (cox1) sequence, a fragment of 650 bp, was obtained for cox1. Four fragments of 409 bp were successfully sequenced and submitted to the GenBank database, with accession numbers OQ799521, OQ799522, OQ799523 and OQ799524.

Etymology: the specific name of this nematode refers to its geographical distribution.

### 3.2. Description

General: The specimens were large, stout worms that had a thick cuticle, with transverse striations. The anterior end featured two highly developed pseudolabia raised above the mouth opening (Figure 2A,B, Figure 4A and Figure 5A–C). Each pseudolabium was formed by a well-developed lateral lobe and two smaller sub-median lobes (Figure 2B, Figure 4A and Figure 5A–C). Each lateral lobe had four tooth-like outgrowths in its internal side (Figure 2B, Figure 4A and Figure 6C). Each sub-median lobe had two tooth-like outgrowths in its internal side (Figure 2B, Figure 4A and Figure 6C). There were four pairs of sub-median cephalic papillae at the base of the sub-median lobes, and two amphids in the lateral lobes (Figure 2B, Figure 4A and Figure 6A,B). The pharynx was thick-walled and laterally compressed (Figure 4A and Figure 5B). The esophagus was divided into anterior muscular and posterior glandular parts (Figure 2A). There was a nerve ring that surrounded the esophagus at the level of its muscular part (Figure 2A). An excretory pore was located slightly posterior to the nerve ring (Figure 2A and Figure 6A). The deirids were found between the nerve ring and excretory pore (Figure 6A). Phasmids were not observed in the total mounts.

In the male specimens (11 specimens measured; range, mean in parentheses, holotype measurements in brackets) (Figure 2C,E, Figure 3A–D and Figure 4A–E), the oral opening was surrounded by two tri-lobed pseudolabia (Figure 4A). There were four tooth-like outgrowths present in the lateral lobes, and two tooth-like outgrowths in the sub-median lobes (Figure 4A). The body length was measured to be 13.67–23.37 mm (18.43 mm) [15.02 mm]; the width at the level of the esophagus base was 330–526 (430) [382]. The length of the pharynx was 39–69 (56) [57]. The muscular esophagus had a length of 219–340 (280) [219]; the width at its base was 75–103 (82) [75]. The glandular esophageal length was 2.95–4.67 mm (3.97 mm) [3.75 mm], and the width at its base was 123–206 (167) [165]. There was a nerve ring located at 123–273 (217) [123] from the cephalic end. The deirids were located at 213–308 (250) [231] from the cephalic end. An excretory pore was located at 371–567 (474) [351] from the cephalic end. The posterior end of the body strongly curved ventrally. The pericloacal surface was ornamented with cuticular formations (Figure 2C, Figure 3A and Figure 4B–E). There was a total of 17 caudal papillae; 4 pairs of large and pedunculated precloacal papillae (pairs 1 to 4) (Figure 2C, Figure 3A and Figure 4B), 1 large unpaired precloacal papilla (Figure 2C, Figure 3B and Figure 4B) and 4 pairs of postcloacal papillae (pairs 5 to 8) (Figure 2C, Figure 3C and Figure 4B,C). The postcloacal papillae in pair 5 were large, pedunculated and placed near the posterior edge of the cloaca (Figure 2C, Figure 3C and Figure 4B,C). The remaining three pairs of postcloacal papillae (pairs 6 to 8) were grouped and placed near the posterior tip (Figure 2C, Figure 3C and Figure 4B,C). The papillae of pair 6 were large and pedunculated (Figure 2C, Figure 3C and Figure 4B,C). The papillae of pairs 7 and 8 were smaller and apparently sessile (Figure 2C, Figure 3C and Figure 4B,C). Phasmids were not observed. The observed spicules were unequal in size and shape, with the right spicule being longer and thinner 643–715 (675) [694] (Figure 2C and Figure 3D); the left spicule was thick and alate, and shorter than the right spicule 309–412 (368) [360] (Figure 2C and Figure 3D). The gubernaculum was V-shaped, 116–159 (141) [123] (Figure 2D).

The observed females (13 specimens measured; range, mean in parentheses, allotype measurements in brackets) (Figure 2A,B,D,F and Figure 5A–C) had an oral opening surrounded by two tri-lobed pseudolabia (Figure 2A,B and Figure 5A–C). There were four tooth-like outgrowths present in the lateral lobes, and two tooth-like outgrowths in the sub-median lobes (Figure 5C). The body length measured as 25.52–41.99 mm (32.51 mm) [27.73 mm]; the width at the level of the vulva was 795–1455 (1137) [815], and the pharynx had a length of 57–90 (74) [87]. The muscular esophagus had a length of 273–464 (346) [310], with a width at its base of 108–175 (128) [113]. The glandular esophageal length was 3.49–5.79 mm (4.52 mm) [3.84 mm], with a width at its base of 165–310 (230) [185]. The nerve ring was located at 234–311 (278) [311] from the cephalic end (Figure 2A). There were deirids located at 273–360 (317) [309] from the cephalic end. The excretory pore was located at 464–660 (577) [309] from the cephalic end (Figure 2A). The vulva was slightly pre-equatorial, and was located at a 11.92–19.43 mm (15.22 mm) [13.33 mm] distance from the cephalic end. The tail was 258–423 (327) [289] (Figure 2D). The embryonated eggs were 48.9–56.7 × 33.4–41.1 (51.8 × 38.2) [51.4 × 38.6] (Figure 2F).

The L3 larva (one specimen measured) (Figure 6A–C) had a body length of 5.91 mm, a width at the level of the esophagus basis of 162. The length of the pharynx was 33. The muscular esophagus had a length of 159, with a width at its base of 39. The glandular esophageal length was 1.62 mm, and the width at its base was 67. The nerve ring was located at 121 from the cephalic end. Deirids were not observed. The excretory pore was located at 270 from the cephalic end, and the anus at 495 from the posterior end. The tail was notched, with 10 pointed outgrowths arranged circularly in the same plan (Figure 6C).

### 3.3. Molecular Phylogeny

All of the genomic DNA samples were amplified to the expected size (650 bp). Four of these amplicons were selected for sequencing, as all of the fragments had the same host species (*R. rattus*) and had the same origin (Frontera, El Hierro, Canary Islands, Spain); this was carried out in order to optimize resources. Four 409-base-pair fragments were successfully sequenced and sent to the GenBank database with accession numbers OQ799521, OQ799522, OQ799523 and OQ799524. All of the sequences showed 97% query coverage values and 84.48% identity with *Protospirura muricola*.

**Figure 7:**
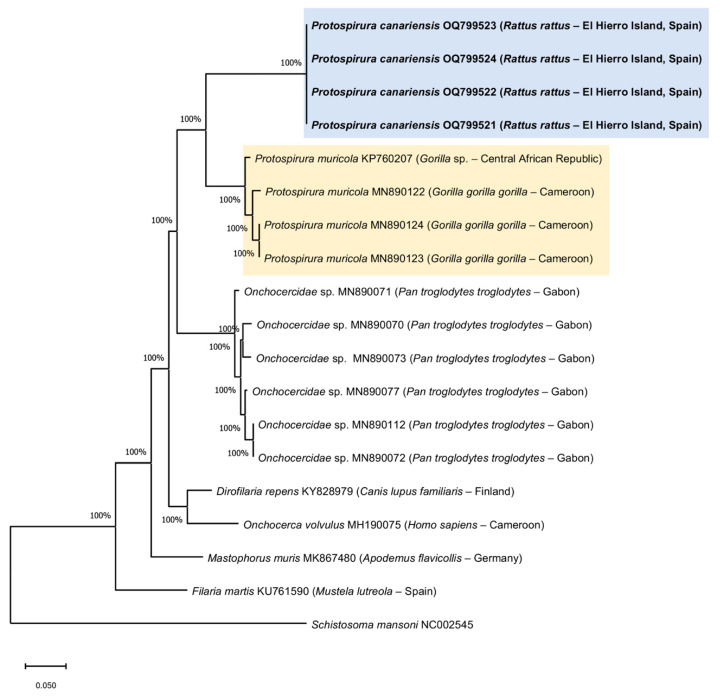
Phylogenetic analysis using the maximum likelihood method with p distance and 1000 bootstrap replications based on the mitochondrial cytochrome c oxidase subunit I gene (cox1) sequence. Sequences obtained in the present study are shown in bold. *Schistosoma mansoni* was used as the outgroup.

Phylogenetic analyses of the cox1 gene showed a tree that was composed of six monophyletic groups (Figure 7). We observed a clade in the upper part that was composed of the specimens of *Protospirura canariensis* n. sp. contributed by this study. Below was a clade made up of the species of *P. muricola* with a robustness of 100%. Both clades were clearly separated from the rest of the included sequences.

## 4. Discussion

One of the most useful morphological characteristics in the recognition and differentiation of *Protospirura* species concerns the morphology of the pseudolabia and their associated structures. The oral opening was surrounded by two pseudolabia, each with three lobes (a larger lateral lobe and two smaller sub-median lobes). In the internal faces of these lobes were tooth-like outgrowths that were variable in number (usually two or four). *Protospirura canariensis* n. sp. has four tooth-like outgrowths in each lateral lobe and two tooth-like outgrowths in each sub-median lobe. Only *P. peromysci* Babero and Matthias, 1967, exhibits a similar arrangement of tooth-like outgrowths in the pseudolabia lobes [3]. However, there are some discrepancies among earlier studies that described several *Protospirura* species, including *P. peromysci* (see Table 1). Thus, Babero and Matthias [3], in the original description of the species, mentioned the presence of four flat denticles in the lateral lobes and two triangular denticles in sub-median lobes, while Smales [9] considered this species to have four teeth in both the lateral and sub-median lobes. In male specimens, *P. canariensis* n. sp. can be differentiated from *P. peromysci* in terms of the size of the right spicule (643–715 µm vs. 820–1200 µm, respectively) and in the number of cloacal papillae (17 in *P. canariensis* n. sp. vs. 23 in *P. peromysci*) [3].

In male specimens, the size of spicules is another diagnostic character to consider in comparing the species of *Protospirura*. Considering the size of both the right and left spicules, *P. pseudomuris* Yokohata and Abe, 1989, and *P. siamensis* present similar-sized spicules: the right spicule measures 643–715 µm in *P. canariensis* n. sp., 540–790 µm in *P. pseudomuris* and 469–701 µm in *P. siamensis*; the left spicule measures 309–412 µm in *P. canariensis* n. sp., 270–380 µm in *P. pseudomuris* and 348–380 µm in *P. siamensis* [7,14]. Both species can be differentiated from the new species in the number of tooth-like outgrowths in the lobes of the pseudolabia (see Table 1). Additionally, the number of cloacal papillae also distinguish the new species from *P. pseudomuris* (17 vs. 21, respectively). In the remaining *Protospirura* species, the spicule sizes are out of the range of those of *P. canariensis* n. sp. (see Table 1).

The number, morphology and disposition of cloacal papillae are other differentiating characters between *P. canariensis* n. sp. And the remaining species of the genus *Protospirura*. Thus, *P. canariensis* n. sp. has a total of 17 cloacal papillae, 4 pedunculated precloacal pairs, an unpaired precloacal papilla located in the front edge of cloaca, and 4 postcloacal pairs (1 pedunculated pair near the cloaca and 1 pedunculated and 2 sessile pairs near the posterior tip). Only two species have a similar number of cloacal papillae; the first of them is *P. mexicana* Falcón and Sanabria, 1995, which has 17 cloacal papillae with a similar arrangement, although Falcón and Sanabria [4] described some variability in the arrangement, particularly for the postcloacal papillae; the second one is *P. siamensis*, with males that have 4 or 5 precloacal pairs of cloacal papillae for a total of 17 or 19 cloacal papillae [14]. Both species clearly differ from *P. canariensis* n. sp. in the number of tooth-like outgrowths present in the sub-median lobes of the pseudolabia (1 in *P. mexicana*, 4 in *P. siamensis* and 2 in *P. canariensis* n. sp.) and in the size of the spicules, particularly the right spicule (340–465 µm in *P. mexicana*, 469–701 µm in *P. siamensis* and 643–715 µm in *P. canariensis* n. sp.). The remaining species of *Protospirura* have a higher number of cloacal papillae, comprising between 18 and 25 papillae (see Table 1).

In the females of *P. canariensis* n. sp., the vulva is located slightly before the mid-body. Other *Protospirura* species, namely *P. kaindiensis*, *P. okinavensis* and *P. pseudomuris*, also present a pre-equatorial placement of the vulva [7,8,9]. Females of the new species differ from those of these three species in the number of tooth-like outgrowths present in the lateral and sub-median lobes of the pseudolabia (see Table 1).

Within the *Protospirura* genus, the life cycles of *P. muricola* and *P. numidica criceticola* Quentin, Karimi and Rodriguez de Almeida, 1968, have been elucidated [13,20,26]. Quentin et al. [13] described the life cycle of *P. numidica criceticola* with an L3 larva characterized by a notched posterior end that had 12 to 15 pointed outgrowths. Posteriorly, Quentin [20] elucidated the life cycle of *P. muricola* and described a L3 larva that presented nine pointed outgrowths arranged in the same plan in its notched posterior end. In the present study, the single recovered L3 larva of *P. canariensis* n. sp. had 10 pointed outgrowths that were arranged circularly in the same plan. This morphological trait differs clearly from the two above-mentioned L3 larva of *Protospirura* [13,20], and is an additional feature that differentiates these three species.

**Table 1:** Some discriminating characteristics of *Protospirura* species.

Species	Pseudolabia		Spicules		Cloacal Papillae	Vulva	References
	Subm	Lat	Right	Left			
*Protospirura anopla* Kreis, 1938	0 [6] 4 [9]	0 [6] 4 [9]	287	632	4 pre, 5 post (18)	posteq	[6,9]
*Protospirura armeniana* Alojan, 1951	2 [6] 4 [9]	2 [6] 4 [9]	620–639	370–411	4 pre, upre, 7 post, upost (24)	posteq	[6,9]
*Protospirura canariensis* n. sp.	2	4	643–715 (675)	309–412 (368)	4 pre, upre, 4 post (17)	preeq	Present study
*Protospirura chabaudi* Vuylsteke, 1964	4 [9] 2 [18]	4 [9] 0 [18]	980	420	4 pre, 5 post (18)	posteq	[9,18]
*Protospirura kaindiensis* Smales, 2001	2	2	450–480	310–330	4 pre, upre, 5 post (19)	preeq	[9]
*Protospirura mexicana* Falcón and Sanabria, 1995	1	0	340–465 (404)	420–527 (481)	4 pre, upre, 4 post (17)	posteq	[4]
*Protospirura munimuniensis* Smales, 2021	2	2	602–603	430–455	4 pre, upre, 6 post (21)	?	[16]
*Protospirura muricola* Gedoelst, 1916	2	2	268–430 (352)	290–501 (411)	4 pre, upre, 6 post (21)	posteq	[15,25]
*Protospirura numidica* Seurat, 1914	3 [5] 4 [9]	3 [5] 4 [9] 4 [13]	830	420	4 pre, upre, 5 post (19)	posteq	[5,9,13]
*Protospirura numidica criceticola* Quentin, Karimi and Rodriguez de Almeida, 1968	?	4	1250	470	4 pre, upre, 7 post (23)	posteq	[13]
*Protospirura okinavensis* Hasegawa, 1990	4	4	600–650 (620)	320–350 (320)	5–6 pre, upre, 4 post (19 or 21)	preeq	[8]
*Protospirura peromysci* Babero and Matthias, 1967	2 [3] 4 [9]	4 [3] 4 [9]	820–1200	330–380	4 pre, upre, 7 post (23)	posteq	[3,9]
*Protospirura pseudomuris* Yokohata and Abe, 1989	1 [7] 2 [9]	1 [7] 2 [9]	540–790 (680)	270–380 (330)	4 pre, upre, 6 post (21)	preeq	[7,8,9]
*Protospirura siamensis* Ribas, Veciana, Chaisiri and Morand, 2012	4	4	469–701 (572.6)	348–380 (368.7)	4–5 pre, upre, 4 post (17 or 19)	posteq	[14]
*Protospirura suslica* Schulz, 1927	2 [6] 4 [9]	2 [6] 4 [9]	315	873	5 pre, upre, 6 post (23)	posteq	[6,9]

Mean size of spicules in parentheses. Total number of cloacal papillae in parentheses. (Lat) tooth-like outgrowths in lateral lobes; (post) postcloacal pairs of papillae; (posteq) postequatorial; (pre) precloacal pairs of papillae; (preeq) pre-equatorial; (Subm) tooth-like outgrowths in sub-median lobes; (upost) unpaired postcloacal papilla; (upre) unpaired precloacal papilla; (?) doubtful or unknown data.

The parasitized host and the geographical distribution are additional criteria that distinguish *P. canariensis* n. sp. from the remaining species of *Protospirura*. There are seven *Protospirura* species parasites of Muridae rodents, namely *P. armeniana*, *P. chabaudi*, *P. kaindiensis*, *P. munimuniensis*, *P. muricola*, *P. okinavensis* and *P. siamensis* [6,8,9,14,15,16,17,18,19]. *Protospirura armeniana* was found to parasitize the house mouse *Mus musculus* Linnaeus, 1758, in Mongolia and Armenia [6,17,19]. *Protospirura chabaudi* was found to parasitize *R. rattus* in the Democratic Republic of Congo [18]. Both *P. kaindiensis* and *P. munimuniensis* were found in Papua New Guinea to be parasitizing *Pseudohydromys murinus* Rümmler, 1934, and *Chiruromys lamia* (Thomas, 1897), respectively [9,16]. *Protospirura okinavensis* was reported in Japan to be parasitizing *Mus caroli* Bonhote, 1902 [8]. *Protospirura muricola* is the species with the widest geographical distribution to be parasitizing Muridae rodents, including in numerous African and Asian countries, and also with the largest host range recorded in diverse genera of murids such as *Arvicanthis* Lesson, 1842, *Gerbilliscus* Thomas, 1897, *Hybomys* Thomas, 1910, *Lemniscomys* Trouessart, 1881, *Malacomys* Milne-Edwards, 1877, *Mastomys* Thomas, 1915, *Mus* Linnaeus, 1758, *Praomys* Thomas, 1915, *Rattus* Fischer, 1803 and *Uranomys* Dollman, 1909 [25,27,28,29,30,31]. Finally, *P. siamensis* was described in Thailand to be parasitizing several murids of genera *Bandicota* Gray, 1873, *Berylmys* Ellerman, 1947, *Mus* and *Rattus* [14], and more recently was found in rats and mice from Pakistan [32]. However, these authors did not specify the species of murids parasitized by *P. siamensis* [32]. Thus, to our knowledge, apart from *P. canariensis* n. sp. presently recorded at the Canary Archipelago, only two species of *Protospirura*, namely *P. chabaudi* and *P. muricola*, have been cited as parasitizing the black rat *R. rattus* and reported in Democratic Republic of Congo, Nigeria, China and Taiwan [18,25,28,29].

The molecular results confirmed the morphological observations that showed that the specimens described in this study are a new species that is clearly separated from the genus *Mastophorus* and cluster with *P. muricola*. The remaining species of the genus *Protospirura* should be re-evaluated by integrating morphologic discriminant characters and molecular information.

In conclusion, the morphological characteristics, such as the pseudolabia and the number of tooth-like outgrowths present in their lateral and sub-median lobes, the laterally compressed pharynx, the particular characters of males (number and arrangement of cloacal papillae, and size of spicules) and females (position of the vulva), the molecular data, and the geographical distribution and the parasitized host, all identify the discovered nematode as being a new species of the genus *Protospirura*.

## 5. Conclusions

The present research contributes with the description of a new spirurid nematode of the genus *Protospirura*, *P. canariensis* n. sp., and provides further information on taxonomic criteria that are useful for the characterization of species in this genus.

The most useful characteristics to differentiate *P. canariensis* n. sp. from the remaining species of the genus *Protospirura* are the number of tooth-like outgrowths present in the lateral and sub-median lobes of the pseudolabia both in males and females; the size of spicules and the number and arrangement of the cloacal papillae in male specimens; and the position of the vulva in female specimens. The host parasitized and the geographical distribution are additional and useful criteria. Thus, the current finding in El Hierro Island (Canary Archipelago, Spain) enlarges the geographical distribution of the genus *Protospirura*. After many years of trapping campaigns in all of the islands of the Canary Archipelago, *P. canariensis* n. sp. has been found only in El Hierro Island, despite the existence of biotopes of similar biotic and abiotic characteristics in other islands of the archipelago. Unlike Tenerife and the Gran Canaria islands, El Hierro is a small island without any commercial seaport, having only connections with the remaining islands through local planes and ferries. This may be the most feasible cause of the presence of the new species only in El Hierro. Finally, the sequence of the subunit I gene of the cytochrome c oxidase (cox1) also characterizes and differentiates *P. canariensis* n. sp. from *P. muricola*, the species with the widest geographical distribution, and one of the two *Protospirura* species found to parasitize *R. rattus*.

## Data Availability

The type specimens are available upon request from MNHN Paris. Additional specimens are available upon request from the corresponding author.

## References

[1] Chitwood B.G., Silva B., de Almeida B.J., Ferreira N., Gonçalves A., Strommer S., Gonçalves O., de Almeida C.A., Cantizano dos Santos A. (1938). The status of *Protospirura* vs. *Mastophorus* with a consideration of the species of these genera. Livro Jubilar do Professor Lauro Travassos.

[2] Wertheim G. (1962). A study of *Mastophorus muris* (Gmelin, 1790) (Nematoda: Spirurida). Trans. Am. Microsc. Soc..

[3] Babero B.B., Matthias D. (1967). *Protospirura peromysci* n. sp. (Nematoda: Spiruridea) and other helminths from *Peromyscus* spp. in Nevada. Proc. Helminthol Soc. Wash..

[4] Falcón Ordaz J., Sanabria Espinoza M.A. (1995). Especie nueva del género *Protospirura* (Nemata: Spiruridae) de *Peromyscus difficilis* (Rodentia: Cricetidae) de Hidalgo, México. An. Inst. Biol. UNAM Ser. Zool..

[5] Seurat L.-G. (1914). Sur un nouveau spiroptère du Chat ganté. C. R. Soc. Biol. Paris.

[6] Skryabin K.I., Sobolev A.A., Skryabin K.I. (1963). Spirurata of Animals and Man and the Diseases Caused by Them. Spirurata, Part 5, Spiruroidea. Osnovy Nematologii.

[7] Yokohata Y., Abe H. (1989). Two new Spirurid Nematodes in Japanese Moles, *Mogera* spp.. Jpn. J. Parasitol..

[8] Hasegawa H. (1990). *Protospirura okinavensis* sp. n. (Nematoda: Spiruridae) from *Mus caroli* on Okinawa Island, Japan. J. Helminthol. Soc. Wash..

[9] Smales L.R. (2001). *Protospirura kaindiensis* n. sp. (Spirura: Spiruridae) and other helminths from *Pseudohydromys* (Muridae: Hydromyinae) from Papua New Guinea. J. Parasitol..

[10] Chabaud A.G., Anderson R.C., Chabaud A.G., Willmott S. (1975). No. 3 Keys to genera of the order Spirurida. Part 2. Spiruroidea, Habronematoidea and Acuarioidea. CIH Keys to the Nematode Parasites of Vertebrates.

[11] Kreis H.A. (1938). Beitrage zur Kenntnis parasitischer nematoden. VII. Parasitische Nematoden der schweizerischen wissenschaftlichen Expedition nach Angola (Afrika) in Jahre 1932. Ctbl. Bakt. I. Orig..

[12] Pence D.B., Windberg L.A. (1984). Population dynamics across selected habitat variables of the helminth community in coyotes, *Canis latrans*, from South Texas. J. Parasitol..

[13] Quentin J.-C., Karimi Y., Rodríguez de Almeida C. (1968). *Protospirura numidica criceticola*, n. subsp. parasite de Rongeurs Cricetidae du Brésil. Cycle évolutif. Ann. Parasitol..

[14] Ribas A., Veciana M., Chaisiri K., Morand S. (2012). *Protospirura siamensis* n. sp. (Nematoda: Spiruridae) from rodents in Thailand. Syst. Parasitol..

[15] Smales L.R., Harris P.D., Behnke J.M. (2009). A redescription of *Protospirura muricola* Gedoelst, 1916 (Nematoda: Spiruridae), a parasite of murid rodents. Syst. Parasitol..

[16] Smales L.R. (2021). The gastrointestinal nematodes of *Chiruromys forbsei* Thomas and *C. lamia* (Thomas) (Rodentia: Muridae) with the description of a new species of *Helgenema* (Heligmonellidae) and a new species of *Protospirura* (Spiruridae) from Papua New Guinea. Trans. R. Soc. S. Aust..

[17] Tinnin D.S., Ganzorig S., Gardner S.L. (2011). Helminths of small mammals (Erinaceomorpha, Soricomorpha, Chiroptera, Rodentia, and Lagomorpha) of Mongolia.

[18] Vuylsteke C. (1964). Mission de Zoologie médicale au Maniema (Congo, Léopoldville) (P.L.G. Bennoit, 1959). 3. Vermes–Nematoda. Ann. Mus. R. Afr. Centr. Ser. 8 Zool..

[19] Movsesyan S.O., Nikoghosian M.A., Petrosian R.A., Vlasov E.A., Kuznetsov D.N. (2018). Nematodes of rodents of Armenia. Ann. Parasitol..

[20] Quentin J.-C. (1969). Cycle biologique de *Protospirura muricola* Gedoelst, 1916 Nematoda Spiruridae. Ann. Parasitol..

[21] López C., Clemente S., Almeida C., Brito A., Hernández M. (2015). A genetic approach to the origin of *Millepora* sp. in the eastern Atlantic. Coral Reefs.

[22] Gaillard C.M., Pion S.D., Hamou H., Sirima C., Bizet C., Lemarcis T., Locatelli S. (2020). Detection of DNA of filariae closely related to *Mansonella perstans* in faecal samples from wild non-human primates from Cameroon and Gabon. Parasites Vectors.

[23] Kumar S., Stecher G., Li M., Knyaz C., Tamura K. (2018). MEGA X: Molecular evolutionary genetics analysis across computing platforms. Mol. Biol. Evol..

[24] Kimura M. (1980). A simple method for estimating evolutionary rate of base substitutions through comparative studies of nucleotide sequences. J. Mol. Evol..

[25] Baylis H.A. (1928). On a collection of nematodes from Nigerian mammals (chiefly rodents). Parasitology.

[26] Anderson R.C. (2000). Nematode Parasites of Vertebrates. Their Development and Transmission.

[27] Brouat C., Kane M., Diouf M., Bâ K., Sall-Dramé R., Duplantier J.M. (2007). Host ecology and variation in helminth community structure in *Mastomys* rodents from Senegal. Parasitology.

[28] Chen H.T. (1933). A preliminary report on a survey of animal parasites of Canton, China, rats. Lignan Sci. J..

[29] Hasegawa H., Kobayashi J., Otsuru M. (1994). Helminth parasites collected from *Rattus rattus* on Lanyu, Taiwan. J. Helminthol. Soc. Wash..

[30] Smales L.R. (2016). The gastrointestinal helminths of *Rattus niobe* (Rodentia: Muridae) with descriptions of two new genera and three new species (Nematoda) from Papua New Guinea and Papua Indonesia. Zootaxa.

[31] Temme M. (1983). Stomach nematodes of the Polynesian rat *Rattus exulans* in the Northern Marshall Islands, Pacific Ocean. Z. Angew. Zool..

[32] Rajper M., Birmani N.A. New record of *Protospirura siamensis* Ribas, 2012 (Nematoda: Spiruridae) recovered from Rat and Mice of district Hyderabad, Sindh, Pakistan. Proceedings of the 40th Pakistan Congress of Zoology.

